# Remodeling of lipid bodies by docosahexaenoic acid in activated microglial cells

**DOI:** 10.1186/s12974-016-0580-0

**Published:** 2016-05-24

**Authors:** Marie-Eve Tremblay, Issan Zhang, Kanchan Bisht, Julie C. Savage, Cynthia Lecours, Martin Parent, Vladimir Titorenko, Dusica Maysinger

**Affiliations:** Department of Molecular Medicine, Faculty of Medicine, Université Laval, Axe Neurosciences, Centre de recherche du CHU de Québec, Québec, QC Canada; Department of Pharmacology and Therapeutics, McGill University, Montréal, QC Canada; Department of Psychiatry and Neuroscience, Faculty of Medicine, Centre de recherche de l’Institut universitaire en santé mentale de Québec, Université Laval, Québec, QC Canada; Department of Biology, Concordia University, Montréal, QC Canada

**Keywords:** Docosahexaenoic acid, Lipopolysaccharide, N9 microglial cells, Microglia, Lipid bodies, Mitochondria, Endoplasmic reticulum, Inflammation, Phagocytosis

## Abstract

**Background:**

Organelle remodeling processes are evolutionarily conserved and involved in cell functions during development, aging, and cell death. Some endogenous and exogenous molecules can modulate these processes. Docosahexaenoic acid (DHA), an omega-3 polyunsaturated fatty acid, has mainly been considered as a modulator of plasma membrane fluidity in brain development and aging, while DHA’s role in organelle remodeling in specific neural cell types at the ultrastructural level remains largely unexplored. DHA is notably incorporated into dynamic organelles named lipid bodies (LBs). We hypothesized that DHA could attenuate the inflammatory response in lipopolysaccharide (LPS)-activated microglia by remodeling LBs and altering their functional interplay with mitochondria and other associated organelles.

**Results:**

We used electron microscopy to analyze at high spatial resolution organelle changes in N9 microglial cells exposed to the proinflammogen LPS, with or without DHA supplementation. Our results revealed that DHA reverses several effects of LPS in organelles. In particular, a large number of very small and grouped LBs was exclusively found in microglial cells exposed to DHA. In contrast, LBs in LPS-stimulated cells in the absence of DHA were sparse and large. LBs formed in the presence of DHA were generally electron-dense, suggesting DHA incorporation into these organelles. The accumulation of LBs in microglial cells from mouse and human was confirmed in situ. In addition, DHA induced numerous contacts between LBs and mitochondria and reversed the frequent disruption of mitochondrial integrity observed upon LPS stimulation. Dilation of the endoplasmic reticulum lumen was also infrequent following DHA treatment, suggesting that DHA reduces oxidative stress and protein misfolding. Lipidomic analysis in N9 microglial cells treated with DHA revealed an increase in phosphatidylserine, indicating the role of this phospholipid in normalization and maintenance of physiological membrane functions. This finding was supported by a marked reduction of microglial filopodia and endosome number and significant reduction of LPS-induced phagocytosis.

**Conclusions:**

DHA attenuates the inflammatory response in LPS-stimulated microglial cells by remodeling LBs and altering their interplay with mitochondria and other associated organelles. Our findings point towards a mechanism by which omega-3 DHA participates in organelle reorganization and contributes to the maintenance of neural cell homeostasis.

**Electronic supplementary material:**

The online version of this article (doi:10.1186/s12974-016-0580-0) contains supplementary material, which is available to authorized users.

## Background

Docosahexaenoic acid (DHA, 22:6n3) is a member of omega-3 polyunsaturated fatty acids. Its physical, chemical, and biological properties were extensively investigated, but its mechanisms of action in neural cells are still unclear since it exerts different biological activities depending on the cell type and physiological or pathological context [1–3]. Several studies suggest anti-inflammatory and immunomodulatory properties of omega-3 long chain polyunsaturated fatty acids including DHA [4]. DHA is proposed to be effective in preventing or treating chronic inflammatory conditions that comprise inflammatory bowel disease, rheumatoid arthritis, and asthma, as well as neurodegenerative disorders [1, 5–7]. DHA is a substrate for the synthesis of lipid mediators such as resolvins, protectins, and maresins, all of which have anti-inflammatory properties [8–10]. DHA is particularly retained and concentrated in the nervous system, where it ameliorates neuronal circuit integrity and plasticity [11] and hence learning and memory functions [1]. A recent meta-analysis in human suggests that DHA, alone or combined with another member of omega-3 polyunsaturated fatty acids, eicosapentaenoic acid (EPA), contributes to improving memory function in older adults with mild memory complaints [12]. We hypothesize that DHA-induced organelle remodeling in microglia activated by the proinflammogen lipopolysaccharide (LPS) contributes to the preservation of microglial cellular functions.

At the cellular level, DHA is mainly incorporated into cell membranes and lipid bodies (LBs). LBs are dynamic, biological micelles composed of a lipophilic core that mainly contain neutral lipids (cholesterol esters and triglycerides) and a phospholipid monolayer surface with numerous associated signaling proteins [13–17]. Among these, the most investigated proteins are perilipins. Several perilipins were found to be associated with different sizes of LBs, playing a crucial role in the regulation of LBs stability and biogenesis [18]. Stabilized LBs could contribute to the maintenance of lipid homeostasis and cell function, whereas inadequate surface protection could lead to inadequate lipid compartmentalization [19]. Large LBs are actively formed in vivo in several immune cells during inflammation and constitute sites for the synthesis and storage of various inflammatory mediators [20]. An emerging view is that the remodeling of LBs could represent an early biomarker of neuroinflammation and modulator of neurodegenerative diseases. In particular, perilipin 2 expression was found to be increased in a time-dependent manner in activated N9 microglial cells and primary microglia, concomitantly with the activation of c-Jun N-terminal kinase (JNK) and the p38 MAPK stress signaling pathways [21–23]. Additionally, it was recently shown that LBs accumulation in microglia and astrocytes, driven by mitochondrial defects and increased oxidative stress in neurons, can promote neurodegeneration [24], but the mechanisms involved are still undetermined.

Beneficial and detrimental contributions of microglia to neuronal circuit integrity and plasticity are associated with their phagocytic ability, depending on their particular phenotype and nature of the phagocytosed elements [25]. Phagocytosis is generally considered beneficial since it eliminates dead cells and induces an anti-inflammatory response. However, it can also activate respiratory bursts resulting in the generation of reactive oxygen and nitrogen species [25]. Additionally, microglia can eliminate viable neurons through “phagoptosis,” an endocytic program of cell death execution [26]. During nervous system development, the phagocytosis of neurons and synaptic elements (pre-synaptic axon terminals and post-synaptic dendritic spines) plays a prominent role in the establishment of brain circuits [27, 28]. In the adult brain, microglia further contribute to maintaining homeostasis through their dynamic surveillance of the parenchyma and to different processes involved in brain plasticity [29–31].

LPS has been often used as a proinflammogen to stimulate microglia and induce an inflammatory response [32]. LPS was also used to determine the effectiveness of anti-inflammatory agents, alone or incorporated into nanodelivery systems [21, 22]. Our recent investigations showed that DHA can attenuate microglial dysfunction and disruption of neuronal circuit integrity in organotypic hippocampal cultures exposed to LPS [22]. In particular, DHA increased LB numbers and prevented mitochondrial impairment in microglial cells. These changes were associated with a reduced loss of dendritic spines [22]. Nevertheless, due to the limited resolution of confocal microscopy, we could not observe structural changes in LBs and their relationship with mitochondria. Dynamic remodeling of LBs in microglia exposed to LPS, DHA, or both could have a significant impact on LBs communication with associated organelles. Close apposition between LBs and mitochondria could preserve mitochondrial health by facilitating the coupling of triglyceride hydrolysis and promote ATP production and contribute to an enrichment of non-peroxidized cardiolipins in mitochondria [33–35]. Cardiolipins are lipid components of mitochondria mainly localized in their inner membranes. However, they can be externalized and as such serve as signals for mitophagy [34]. Peroxidized cardiolipins have deleterious effects on mitochondrial functions [35–37].

The objective of this study was to characterize organelle remodeling as a mode of DHA-induced neuroprotection in LPS-stimulated N9 microglial cells. Transmission electron microscopy (TEM) was performed to analyze structural changes of LBs, mitochondria, and phagosomes in LPS-stimulated microglia exposed to DHA. High spatial resolution TEM enabled us to reveal unexpected changes in LB organization and their contacts with mitochondria and other associated organelles under employed experimental conditions. Organelle reorganization was particularly marked in activated microglial cells treated with DHA, and it was related to the regulation of inflammation biomarkers and normalization of phagocytosis. The accumulation of LBs in microglia in situ was also confirmed in mouse and human. Furthermore, lipidomic analyses of N9 microglial cells revealed that phosphatidyl serine (PS) was markedly increased by DHA, suggesting its contribution to the preservation of microglial cellular functions. Collectively, our findings show that DHA plays an important role in the remodeling of LBs and associated organelles in cultured microglia, thereby contributing to neural cell homeostasis.

## Results

Our working hypothesis was that DHA attenuates the inflammatory response in LPS-stimulated microglial cells, through the remodeling of LBs and their interplay with mitochondria and other associated organelles.

### Remodeling of lipid bodies

Considering the limited resolution of confocal microscopy, we conducted TEM experiments to provide insights into the combined effects of LPS and DHA on the dynamic remodeling of LBs. Our ultrastructural analyses were conducted at a DHA concentration of 50 μM, since LPS added to the DHA-induced cytotoxicity at higher concentrations. Annexin V binding was significantly increased when N9 microglial cells were exposed to 100 μM DHA for 24 h. High DHA concentrations induced some necrotic cell death in addition to apoptosis, as revealed by propidium iodide-labeled nuclei and fragmented cells (Additional file 1: Figure S1A-C).

**Fig. 1:**
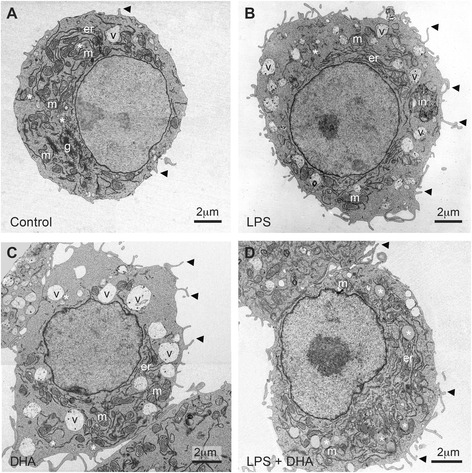
Changes in microglial cell morphology and accumulation of lipid bodies following treatment with LPS, DHA, or a combination of LPS and DHA. **a** In the control condition, microglial cell showing a typical morphology charactrized by few filopodia (*arrowhead*), lipid vacuoles (*v*), and droplets (*asterisk*). *g* Golgi apparatus, *er* endoplasmic reticulum, *m* mitochondria. Vacuoles are recognized by their irregular contours and heterogeneous contents. Droplets are characterized by the roundness of their profiles and uniformity of their contents. **b** Microglial cell in the LPS condition with numerous filopodia and lipid vacuoles but only a few droplets. A phagocytic inclusion (*in*) identified as an accumulation of partially digested membranes can also be seen. **c** Microglial cell exposed to DHA showing several filopodial extensions and an increased prevalence of lipid vacuoles and droplets compared with microglial cell in the control and LPS conditions. In the presence of DHA, lipid droplets with an electron-lucent (*clear*) or electron-dense (*dark*) content and grouped one to another are observed and become the most prevalent form of lipidic inclusion in the combined presence of LPS and DHA (**d**)

**Fig. 2:**
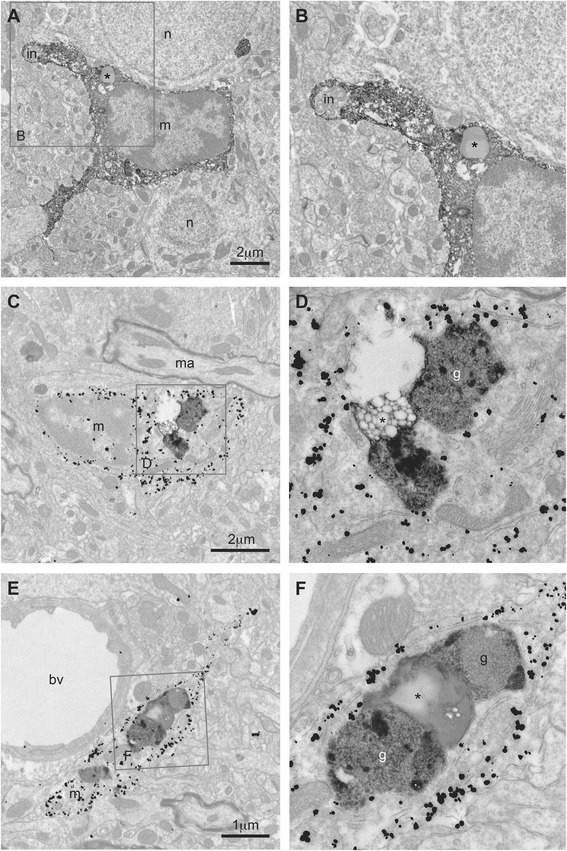
Lipid bodies observed in situ in adult mouse microglia. **a** Microglia (*m*) stained for IBA1 with immunoperoxidase that is observed in direct juxtaposition with two neurons (*n*). A protruding process contains a cellular inclusion (*in*) and a large LB resembling a droplet, in the hippocampus CA1 of an adult mouse (3 months old). **b** *Inset* showing at higher magnification the cellular inclusion, which contains an accumulation of cellular membranes in the process of being digested and, the LB, which displays two electron densities suggesting different lipid compositions. **c** Microglial cell (*m*) stained for IBA1 with immunogold that contains several lipidic inclusions, among which a group of small lipid droplets (*asterisk*) juxtaposing two electron-dense profiles of lipofuscin granules (*g*), in the cerebral cortex of an adult mouse (3 months). *ma* myelinated axon. **d** *Inset* showing at higher magnification the ultrastructural features and relationships between lipid vacuoles and lipofuscin granules. **e** Microglial process (*m*) stained for IBA1 with immunogold, observed in the same mouse, exhibiting several additional examples of lipidic inclusions. **f** *Inset* showing at higher magnification the inclusions: two profiles of lipofuscin granules surrounding an accumulation of very small lipid droplets (*asterisk*) grouped one to another. *Different shades of gray* can be noted among the lipid bodies, suggesting that they contain different lipid species. *bv* blood vessel

We characterized the organization of LBs in N9 microglial cells exposed to LPS proinflammogen stimulation, with or without supplementation with DHA (Fig. 1a–d). Two types of LBs were encountered across our experimental conditions: lipid vacuoles delimited by a bilayer and recognized by their irregular contours and heterogeneous contents, as well as lipid “droplets” or bodies (similar to those described in adipocytes [38] and also found in non-adipocytes [39]) delimited by a monolayer and characterized by their small size, the roundness of their profiles, and the uniformity of their lipid contents [40, 41]. Microglial accumulation of LBs showing various sizes and electron densities was confirmed in situ in C57Bl/6 mouse hippocampus and cerebral cortex (Fig. 2a–f, Additional file 2: Figure S2A), as well as in human hippocampus (Additional file 2: Figure S2B, C and Additional file 3).

**Fig. 3:**
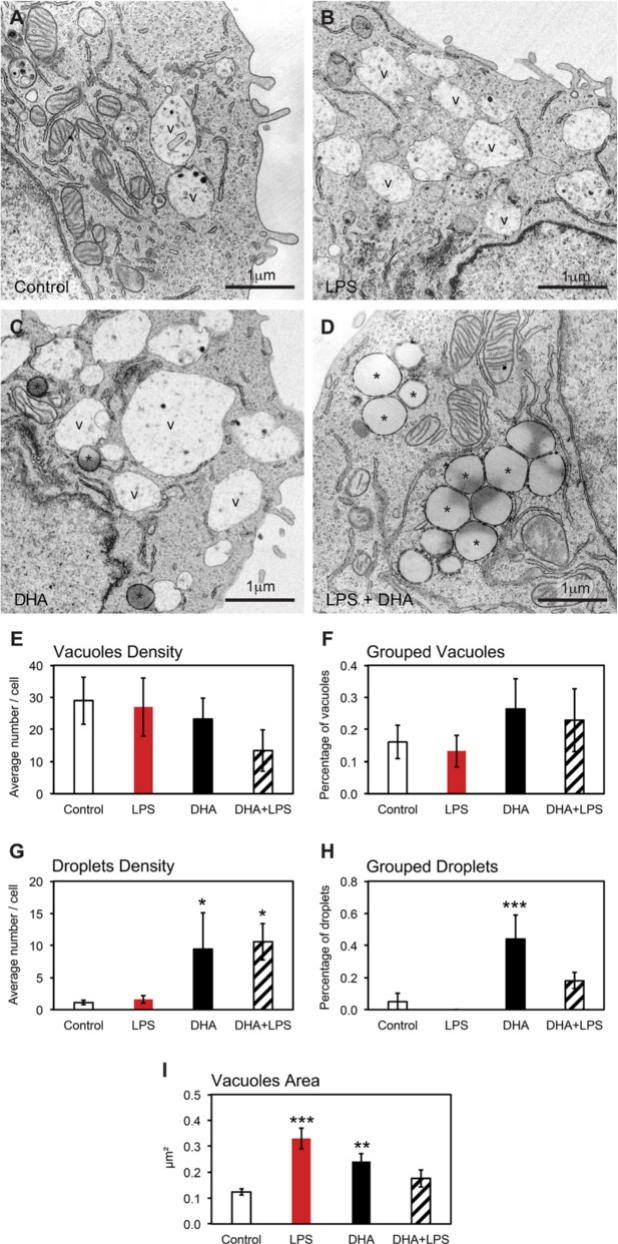
High magnification of lipid bodies in microglial cells following treatment with LPS, DHA, or a combination of LPS and DHA. Few lipid vacuoles (*v*) displaying irregular contours and frequent membranous contents are observed under control conditions (**a**). They become more numerous following treatment with LPS (**b**) and even more following treatment with DHA (**c**). In the presence of DHA, droplets showing round profiles and uniform lipid contents, often with a dark appearance (*asterisk*), also accumulate inside of the microglial cells (**c**). In the combined presence of LPS and DHA, lipid vacuoles become infrequent. Instead, droplets either displaying a clear or a dark appearance, and generally grouped one to another, become the most prevalent from of lipidic inclusion encountered (**d**). **e**–**i** Quantitative analysis of lipid vacuoles and droplets in N9 microglial cells from the four experiments groups. **e**, **g** Average number of lipid vacuoles (**e**) or droplets (**g**) per N9 microglial cell ± SEM. **f**, **h** Average proportion of these lipid vacuoles (**f**) or droplets (**h**) found within groups, i.e., in direct apposition with at least another vacuole or droplet, ±SEM. Mixed groups were sometimes observed. **i** Average size of the lipid vacuoles in N9 microglial cells ± SEM. *p < 0.05, **p < 0.01, and ***p < 0.001

Our analysis in N9 microglial cells revealed that LBs mainly display ultrastructural features of lipid vacuoles under control, LPS, or DHA conditions, while fewer lipid vacuoles were observed in the combined presence of LPS and DHA (Fig. 3a–e). Variations in the size of these lipid vacuoles were noted, displaying smaller sizes in the control condition, medium sizes in the DHA condition, and larger sizes in the LPS condition (Fig. 3i), which confirm the previous observations from confocal microscopy. Additionally, the size of lipid vacuoles was normalized by DHA treatment in the LPS condition (Fig. 3i). Lipid droplets were rarely observed in the control or LPS conditions, where they invariably showed an electron-lucent (clear) content (Fig. 3a, b). Treatment with DHA greatly increased the number of lipid droplets, which were generally small and often showing an electron-dense (dark) content (Fig. 3c, g), suggesting the incorporation of DHA having a high affinity for osmium tetroxide, a lipid fixative used in our cell preparation for electron microscopy [40].

The combined exposure to LPS and DHA further increased the prevalence of lipid droplets in microglial cells (Fig. 3d, g). These lipid droplets often displayed an electron-dense content and were more often found in direct juxtaposition one with another upon DHA treatment (Fig. 3h; contrary to the grouping of vacuoles which remained unchanged; Fig. 3f). Interestingly, a large number of very small and grouped LBs was seen only when microglial cells were exposed to DHA and was detectable by TEM and not by confocal microscopy (Fig. 3d). Overall, these observations indicate that the increase in LBs size and density that is observed with confocal microscopy upon treatment with DHA is accompanied by LBs reorganization and aggregation. This remodeling of LBs could be mediated by the activation and phosphorylation of the mitogen-activated protein kinase p38, as suggested previously [21, 22, 42], considering that activation of p38 and JNK stimulates the formation of LBs in human monocytes [43].

Similarly, very small LBs were observed in situ in mouse and human microglia (Fig. 2c–f and Additional file 2: Figure S2B, C). The very small droplets were often grouped and positioned close to each other and sometimes juxtaposed very large lipofuscin granules (Fig. 2c–f) visible with immunogold that produces a discrete precipitate, unmasking intracellular organelles and lipidic inclusions contrary to immunoperoxidase staining, which diffuses throughout the subcellular compartments. Lipofuscin granules contain several species of fatty acids [44], accumulate during oxidative stress and aging [45], and could interfere with several metabolic processes [46].

### Consequences of LBs association with other organelles

#### Mitochondria

**Fig. 4:**
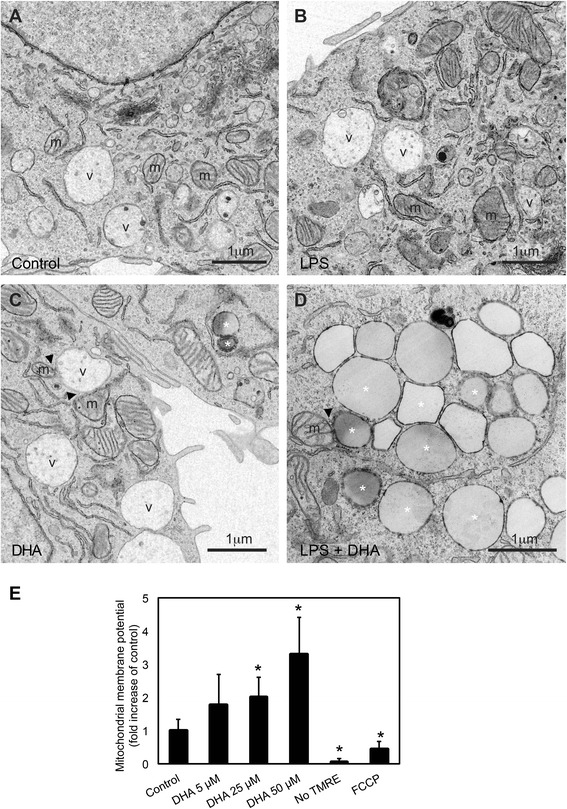
High magnification of direct contacts between lipid bodies and mitochondria in microglial cells, as well as changes in mitochondrial membrane potential following treatment with LPS, DHA, or a combination of LPS and DHA. Few contacts between mitochondria (*m*) and lipid vacuoles (*v*) are observed in the control condition (**a**), and similar observations can be made following treatment with LPS (**b**). In the presence of DHA (**c**) and combined presence of LPS and DHA (**d**), however, direct contacts between mitochondria and lipid bodies, especially lipid vacuoles following DHA treatment and lipid droplets (asterisk) following LPS and DHA treatment, become more prevalent. **e** Changes in mitochondrial membrane potential of N9 microglial cells treated with DHA (5–50 μM) for 24 h. Following treatment, cells were incubated with TMRE (50 nM) for 20 min, after which the media was refreshed and cells were imaged under a fluorescent microscope. Four fields were imaged per treatment, and two cells were imaged per field. Shown are average fold increase in intracellular relative fluorescence intensities ± SEM as compared to untreated control (set to 1) from three independent experiments. Cells imaged in the absence of TMRE and cells treated with FCCP were negative controls. *p < 0.01

LBs remodeling could influence their spatial relationship with mitochondria and their ability to preserve mitochondrial function. Our electron microscopy analysis in microglial cells confirmed that LBs and mitochondria are in close proximity but we found no evidence for the organelle fusion (Fig. 4a–d). In addition to close apposition of LBs with mitochondria, TEM revealed differences in the intimate relationships between lipid vacuoles and mitochondria. In particular, few contacts between mitochondria and lipid vacuoles were observed in microglial cells under control conditions and with LPS treatment (Fig. 4a, b). Treatment with DHA alone resulted in direct contacts between LBs and mitochondria (Fig. 4c), while a combined treatment with DHA and LPS induced numerous direct contacts between the two organelles (Fig. 4d).

Following exposure to LPS, the changes in LBs association with mitochondria were accompanied by a frequent disruption of mitochondrial integrity (Additional file 4: Figure S3A–F). Microglial cells treated with LPS occasionally contained mitochondria partially to completely devoid of cristae and partially to completely devoid of a double membrane, confirming our previous findings that DHA rescues mitochondrial health [22]. In the presence of LPS only, microglial cell mitochondria were also more heterogeneous in size, varying from small to large, while budding of smaller mitochondria from larger ones was sometimes encountered (Additional file 4: Figure S3A–F), indicating an increased remodeling which could be mediated by the activation and phosphorylation of p38 [43]. Abnormalities in mitochondrial morphology are often associated with peroxidation of cardiolipins, specific mitochondrial lipids [47]. Biochemical and morphological abnormalities in mitochondria lead to their functional impairment.

For example, decreased mitochondrial membrane potential is associated with the production of reactive oxygen species [48, 49]. We measured the incorporation of tetramethylrhodamine ethyl ester (TMRE) into N9 microglial mitochondria upon exposure to DHA (5–50 μM DHA) for 1 to 24 h. DHA increased TMRE incorporation in a dose- and time-dependent manner suggesting enhancement of mitochondrial function (Fig. 4e and Additional file 5: Figure S4A–B). This TMRE incorporation was reduced in the presence of LPS, and treatment with DHA normalized it (Additional file 5: Figure S4A, B). DHA additionally decreased the fluorescence intensity of CM-H2DCFDA, a general indicator of reactive oxygen species, both in the presence and absence of LPS, suggesting a reduction of oxidative stress under basal and inflammatory conditions (Additional file 6: Figure S5). Reactive oxygen species cause damage to DNA, proteins, and lipids, may result in a progressive loss of cellular functions, and lead to apoptosis and various pathological conditions.

#### Endoplasmic reticulum

**Fig. 5:**
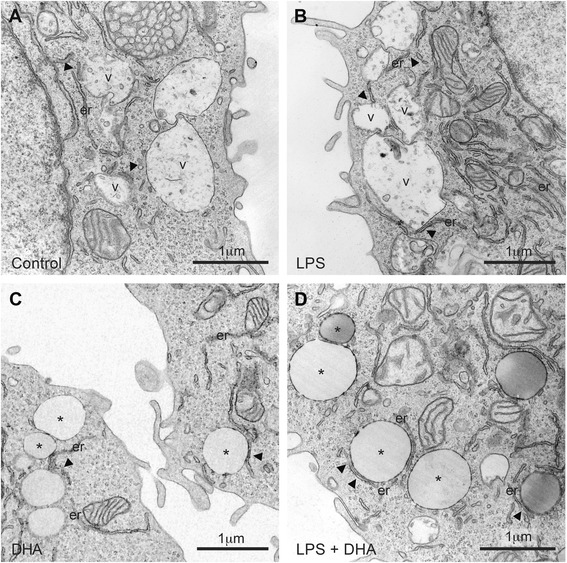
Relationship between lipid bodies and endoplasmic reticulum cisternae in microglial cells at ultrastructural level following treatment with LPS, DHA, or a combination of LPS and DHA. Focal contacts between lipid vacuoles (*v*) and endoplasmic reticulum cisternae (*er*) are observed (*arrowheads*) under control conditions (**a**), as well as following treatment with LPS (**b**). Following treatment with DHA (**c**) or treatment with both LPS and DHA (**d**), stretches of endoplasmic reticulum cisternae are often observed in close proximity (*arrowhead*) with the lipid droplets (*asterisks*) showing a clear or a dark appearance. Long stretches of endoplasmic reticulum cisternae directly touching and surrounding the lipid droplets are particularly observed in the combined presence of LPS and DHA (**d**)

LBs have been shown to interact with the endoplasmic reticulum (ER) across a variety of mammalian cell lines [14, 16]. Nevertheless, the consequences of LBs remodeling on their association with the ER are still undetermined. Here, we used TEM analysis to provide insights into the effects of DHA treatment on the interplay between LBs and the ER in microglial cells. Our analysis revealed only few focal contacts between lipid vacuoles and stretches of ER in the control and LPS-treated cells (Fig. 5a–d). Treatment with DHA changed this association. In particular, the stretches of ER were more often observed in close proximity with the LBs, whether displaying a clear or dark content. The most striking change was observed in microglial cells exposed to LPS and DHA. Their stretches of ER ran parallel to the LBs and intermingled over long distances (Fig. 5d), suggesting the occurrence of functional interactions. In addition, dilation of the ER lumen, which is the best characterized sign of oxidative stress at the ultrastructural level [50], was observed in the LPS-only condition but seldom in other treatment groups. The beneficial effects of DHA on ER health are supported by the previous evidence that DHA contributes to preventing oxidative stress through the restoration of calcium homeostasis in the ER lumen, as required for the proper folding of proteins [51].

**Fig. 6:**
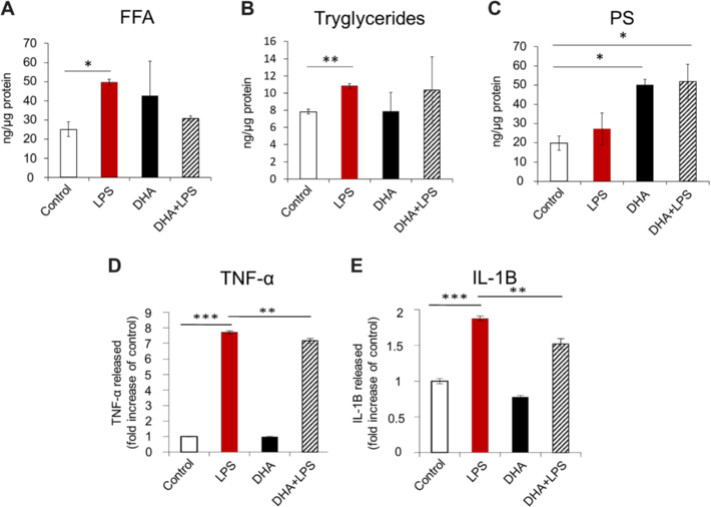
DHA-induced changes in functional biomarkers in activated microglial cells. Mass spectrometric analyses of free fatty acids (*FFA*, **a**) and triglycerides (*TG*, **b**) did not reveal marked changes in the content of these lipids in N9 microglial cells treated with DHA. In contrast, phosphatidyl serine (PS) was significantly increased (**c**). The amount of released proinflmmatory cytokines (**d**–**e**) was moderately but significantly reduced with DHA supplemented in the medium. *p < 0.05, **p < 0.01, and ***p < 0.001. All measurements were performed in triplicate samples in at least two independent experiments

**Fig. 7:**
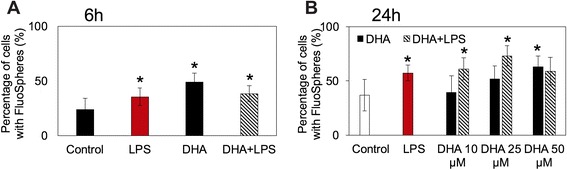
DHA-induced changes in microglial cell phagocytosis. **a**–**b** Effect of DHA on N9 mouse microglial phagocytic activity with and without LPS. N9 microglial cell were cultured for 6 h (**a**) in the presence or absence of DHA (50 μM) and LPS (100 ng/mL) or for 24 h (**b**) in the presence or absence of DHA (10, 25, 50 μM) and LPS (100 ng/mL). FluoSpheres (10^6^ particles/mL) were added to the cells 3 h before the end of treatment. Cells were fixed using 4 % paraformaldehyde and labeled with Hoechst 33258 (10 μM, 10 min). Cells were mounted on glass slides and imaged under a fluorescent microscope. The number of FluoSpheres per cell was counted manually. Shown are average percentages of total cells containing FluoSpheres ± SEM from three independent experiments. *p < 0.01

Our data from mass spectrometric quantification of microglial cells exposed to LPS and/or DHA show that free fatty acids and triglycerides are enhanced with exposure to LPS but not with DHA whereas PS is significantly increased in response to DHA (Fig. 6a–c). PS is the most abundant negatively charged phospholipid in eukaryotic membrane [49]. It also plays a role in signal transduction [50]. Concentrations of generally more abundant phospholipid membrane constituents (phosphatidylcholine, phosphatidylethanolamine, and phosphatidylinositides) were not altered in DHA-treated cells. Interestingly, there was a significant increase in phagocytosis in microglial cells exposed to DHA alone contrary to the bovine serum albumin (BSA) carrier (Fig. 7a, b and Additional file 7: Figure S6); correlating with increased intracellular PS concentrations. This effect was concentration-dependent showing maximal effects at 50 μM, and differences relative to untreated controls were measured as early as 6 h after DHA treatment (50 μM DHA, Fig. 7a, b). Cell treatment with DHA (5–50 μM) for 6 h only did not cause any significant cell death (Additional file 1: Figure S1A–C). Another microglial function affected by DHA treatment was the release of pro-inflammatory cytokines. Both TNFα and IL-1β were significantly increased with LPS treatment and reduced with DHA (Fig. 6d, e). Collectively, these findings suggest that proper incorporation of PS into the inner leaflets of cellular membranes contributes to the intracellular signaling required for the maintenance of cellular integrity and health [52]. In contrast, PS exposed to the cell surface plays a critical role in macrophage recognition of neural cells to be internalized [26, 53].

#### Endosomes

Large endosomes containing an accumulation of heterogeneous contents were also mainly observed in LPS-stimulated microglial cells, sometimes in direct contact with LBs (see Fig. 1b for an example). These elements displayed various signs of digestion, indicating an ongoing state of degradation (Fig. 1 and Additional file 4: Figure S3E, F). Within the endosomes, membranes showing a concentric organization similar to multilamellar bodies, which are lysosomal organelles containing multiple concentric membrane layers, as found in various cell types where their principal functions are storage and secretion of lipids [54], were also often encountered. The autophagic or phagocytic origin of these endosomes is undetermined, especially considering that primary microglia and N9 cells can phagocytose various types of debris, in addition to apoptotic cells in culture. Multilamellar bodies have been shown to be generated through autophagy [55], a catabolic process aimed at recycling cellular components and damaged organelles that is especially triggered by exposure to reactive oxygen and nitrogen species [56].

### Consequences of organelle remodeling on microglial cell functions

Our recent studies showed a marked loss of dendritic spines in hippocampal CA1 region when hyperactive microglial cells produced enhanced amounts of cytokines (including IL-6 and TNFα) and nitric oxide (NO) [57], suggesting that microglial phagocytosis could be implicated in their elimination. In these studies, however, no detailed information on organelle remodeling was provided because of the limited resolution of confocal microscopy.

Concentration- and time-dependent experiments with LPS-stimulated N9 microglial cells revealed a remarkable increase in phagocytic activity within 6 h of treatment (Fig. 7a). Low concentrations of DHA (10 and 25 μM) in the presence of LPS also increased phagocytosis, whereas higher concentrations of DHA (50 μM) attenuated it (Fig. 7b). At the ultrastructural level, this excessive phagocytosis triggered by LPS is supported by the observation that filopodia protruding from N9 microglial cells were most abundant following LPS treatment (Fig. 1b). Filopodia are considered to be cytoplasmic “microspikes” on the leading edge of lamellipodia. They can act as phagocytic tentacles for the engulfment of tissue components by macrophages [58] and microglia [59, 60]. Our functional data together with the electron microscopic analyses of organelle remodeling support the notion that DHA reduces the exacerbated microglial phagocytosis and that restoration of neural cell homeostasis involves a complex dynamic microglial reorganization globally detectable by live cell imaging and at the ultrastructural level [32].

## Discussion

**Fig. 8:**
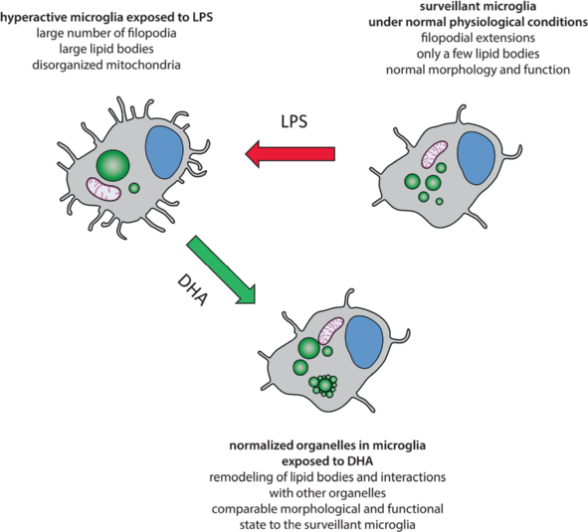
A model of DHA-induced organelle remodeling in LPS-stimulated microglial cells. Microglia under normal physiological conditions are in the surveying state with constantly extending-retracting filopodia and such microglia have only a few lipid bodies (surveying microglia, physiological conditions). Upon exposure to pro-inflammatory agents (e.g., lipopolysaccharide (*LPS*) produced by Gram-negative bacteria) microglia become activated and show transiently an enhanced phagocytosis (e.g., of fluorescent beads or cellular fragments). Such highly activated state is characterized by large number of filopodia and small number of enlarged lipid bodies together with impaired mitochondrial morphology and function. During aging and protracted LPS stimulation, microglial cells gradually lose their protective phagocytic ability. DHA in LPS-hyperactivated microglia normalize the number of filopodia, morphology, and function of mitochondria and lipid bodies thereby maintaining their homeostasis. A particular feature of DHA-induced organelle remodeling is fragmentation of excessively large lipid bodies and facilitation of their association with other organelles (e.g., mitochondria)

The studies show DHA-induced structural organelle remodeling and associated improved functions in the proinflammogen-stimulated mouse N9 microglial cells. Morphological changes of organelles proposed to interact with LBs were revealed using TEM. DHA supplementation increased the prevalence of direct contacts between LBs and mitochondria. DHA also rescued mitochondria from loss of cristae and functional integrity induced by LPS treatment. Based on the data from these and earlier studies, a working model of organelle reorganization in hyperactive microglia exposed to DHA is proposed (Fig. 8).

DHA treatment induces LBs remodeling: the apparent “large” LBs previously detected by confocal microscopy are actually a conglomerate of many small ones. The relevance of this finding is that the mobility and recruitment of proteins to the surface of LBs depends on LB size and is markedly different between the small and big ones [20]. In consequence, the existence of many small LBs with DHA treatment could modify intracellular protein trafficking and docking in microglia and likely in other cell types. The cell type-dependent LB formation was recently shown to play a role in neurodegeneration [23, 24]. A remarkable heterogeneity of LBs and their reversibility was reported in hepatocytes [49] and in vivo in adipocytes [61]. It is conceivable that the heterogeneity is necessary to achieve cell protection by engaging only certain populations of LBs. This could explain the protective role of LBs in DHA-treated cells.

Most of PS molecules are located within the inner cytoplasmic membrane in healthy cells under normal physiological conditions. Soluble cations, polycations and proteins with clustered positively charged domains are attracted to the PS negative charges in the inner leaflets. Such PS transmembrane distribution has implications in PS-mediated intracellular signaling. For instance, when some pathogens cause depletion of PS from phagosomes, the Src tyrosine kinase is also lost [62]. Another example is Akt, a signaling protein with pleckstrin homology domain which interacts with PS. DHA engages signaling pathway via PI3K and Akt activation. Basic residues of Akt are involved in binding to PS and could contribute to DHA-induced Akt activation [63]. We propose that PS species are rapidly transported from the ER to the plasma and endosomal membranes where they may cause specific changes in the membrane biophysical properties (such as in fluidity, thickness, protein-protein, and protein-lipid interactions). This in turn, could attenuate the signaling promoted by LPS- and/or age-related neuroinflammation and neurodegeneration. Moreover, the molecular species of PS induced by DHA may also facilitate the binding of anti-inflammatory cytokines (such as IL-10) to the membrane and its stabilization.

In contrast, DHA reduces the pro-inflammatory cytokines IL-1β and promotes resolution of inflammation [8]. The mechanisms underlying these protective processes are not fully understood. We propose that LBs fragmentation can contribute to these protective effects by favorable lipid segregation, protein recruitment, and increased intracellular mobility. LBs were shown to sequester polyunsaturated fatty acids away from plasma membranes, thereby shielding these lipids from harmful peroxidation [48]. This is especially relevant under conditions of oxidative stress where exogenous supply of DHA can prevent or at least reduce an overall risk of lipotoxicity (e.g., in type 2 diabetes [64]). In addition, DHA-derived resolvin D1 was reported to reduce ER stress-induced apoptosis in hepatic cells [65] and resolvin D1 and E1 to decrease LPS-induced TNFα, IL-6, and IL-1β gene expressions in cultured microglial cells. These findings suggest resolution of cellular inflammatory activity [66]. In macrophages, DHA also decreased the ratio of pro-inflammatory leukotriene B4 to proresolving lipoxin A4, thereby tempering inflammation [67].

LPS-induced mitochondrial defects and increased oxidative stress in neurons could be a missing link between oxidative stress and inflammation leading to neurodegeneration [24]. Formation of excessively large LBs and release of TNFα, IL-1β, and NO from LPS-stimulated N9 microglial cells could exacerbate mitochondrial dysfunction and ER stress in a cell-autonomous or non-autonomous manner. Indeed, an interesting heterogeneity between microglial cells was observed in our cultures. For instance, some LPS-stimulated N9 microglial cells were completely devoid of LBs, even when they were directly juxtaposed to microglial cells containing many LBs (see Additional file 4: Figure S3A for an example). Considering that our TEM studies captured the cells at one time point, time lapse experiments by employing super-resolution microscopy are required to establish the intercellular communication and possible LBs transfer from one cell to another. Organelle heterogeneity and remodeling was also observed with mitochondria, ER, and autophagic vacuoles, between cells, and between different organelles of individual cells. This cellular heterogeneity could be in part explained by different stages in cell cycle. For example, we have evidence for nuclear budding and fragmentation, suggesting an ongoing mitosis and proliferation in a subset of N9 microglial cells exposed to LPS (Additional file 8: Figure S7).

LPS- and DHA-induced organelle remodeling, regulation of cytokine production, and phagocytosis are not necessarily mutually exclusive. For example, excess of pro-inflammatory cytokines production induces LBs formation. Size and composition of LBs can influence phagocytosis. Dysregulated phagocytosis can promote cytokine production, and cytokines can modify mitochondrial and LBs remodeling. It was shown that DHA effects are time- and concentration-dependent [22, 68, 69]. Similarly, LPS-induced microglial activation is time- and concentration-dependent [21]. Our findings at 24 h suggest that DHA has attenuated pro-inflammatory cytokine production, moving microglia towards many of the M2-like states accompanied by changes in their functional status, including the ability to phagocytose. Excessive and defective phagocytosis could lead to “frustrated resolution” and abnormal remodeling of several organelles, including LBs and mitochondria [70]. We suggest that organelle remodeling in association with increased pro-inflammatory cytokine and ROS production induced by LPS are disrupting homeostasis, which in vivo could contribute to CNS impairments during aging. Adequate DHA concentrations are essential for the maintenance of homeostasis of the CNS. Each tissue and even cell type likely requires an optimal DHA concentration to maintain a balance between cytokine production and elimination (phagocytosis) of damaged organelles or misfolded and aggregated proteins. DHA concentrations in our studies were within the concentration range used by others [68]. The DHA metabolite resolvin D1 (RvD1) markedly promotes resolution of inflammation, macrophage polarization towards M2-like phenotypes and promotes nonphlogistic phagocytosis in macrophages [71]. Data from our current studies and those by others [11, 72] suggest that DHA and its metabolite RvD1 are desirable endogenous products mediating resolution. Resolvin D1 upregulates several micro RNAs (miRNAs; e.g., miR-146, miR-219, miR-208) that are involved in NFκB and IL-10 expression in resolution. Fullerton and Gilroy provide a current view on the key players and factors that regulate inflammation and resolution, highlighting the efforts to target pro-resolution pathways in diseases involving inflammation [70]. Clearly, it is not sufficient to achieve full resolution with only one “pro-resolution” drug. Components of the resolution process include organelle remodeling, homeostasis of pro- and anti-inflammatory cytokines, and functions of microglia, one of which is phagocytosis. Phagocytosis is a housekeeping process in many cells that can be induced by an acute exposure to LPS. Some studies show that the LPS derivative monophosphoryl lipid A (MPL) induces microglial phagocytosis in the absence of inflammation [73]. Interestingly, inhibition of microglial phagocytosis has been shown to prevent inflammatory neuronal death [74] and synaptic loss in aging and Alzheimer’s disease in vivo [75, 76].

Collectively, organelle remodeling as we observed in microglial cells in culture likely occurs in vivo in animals and humans. Organelle remodeling under pathological conditions [29, 77] could contribute to synaptic loss and cognitive impairment [78, 79]. The loss of synapses has recently emerged as the best pathological correlate of cognitive decline during aging and neurodegeneration [80–82]. Recent genome-wide association studies have linked polymorphisms in inflammation-related genes to increased risk for Alzheimer’s disease, supporting the conclusion that inflammation can be a causative factor in the disease pathology [83]. Interestingly, in a mouse model of accelerated aging where only neurons undergo DNA damage (accompanied by oxidative stress), microglia display an exaggerated response to LPS in terms of pro-inflammatory cytokines and reactive oxygen species production, as well as phagocytosis [84, 85]. An exacerbated microglial phagocytosis of synaptic elements was recently observed in a mouse model of chronic unpredictable stress [86]. Chronic stress is well known to accelerate aging, predispose and exacerbate progression and symptoms in neurodegenerative diseases [40, 87].

## Conclusions

Results presented in this study suggest that DHA supplementation contributes to the preservation of microglial cell functions by inducing remodeling of their LBs and their direct contacts with mitochondria displaying increased ultrastructural integrity and intermingling with ER cisternae showing reduced lumen dilation. DHA supplementation also increases the levels of PS and modulates phagocytosis. It will be important in future studies to determine the time-course of DHA actions on microglia in vivo [88] and their functional relevance to stress resilience, healthy aging, and cognitive improvement in patients suffering from neuropsychiatric and neurodegenerative diseases.

## Methods

### Animals

All experiments were approved and performed under the guidelines of the Institutional animal ethics committees, in conformity with the Canadian Council on Animal Care guidelines as administered by the Animal Care Committee of Université Laval. The animals were housed under a 12-h light-dark cycle at 22–25 °C with free access to food and water. Three-month-old (*n* = 4) male mice on a C57Bl/6 background were examined in this study for the purpose of validating microglial accumulation of LBs observed in vitro. They were anesthetized with sodium pentobarbital (80 mg/kg, i.p.) and perfused through the aortic arch with 3.5 % acrolein followed by 4 % paraformaldehyde. Transverse sections of the brain (50-μm thick) were cut in sodium phosphate buffer (PBS; 50 mM at pH 7.4) using a vibratome [89].

### Brain section staining and processing for electron microscopy

Sections were washed in PBS, quenched with 0.3 % H_2_O_2_ in PBS for 5 min and then with 0.1 % NaBH_4_ for 30 min at room temperature (RT), washed in Tris-buffered saline (TBS; 50 mM at pH 7.4) containing 0.01 % Triton X100, and processed freely floating for immunostaining. Briefly, sections were pre-incubated for 1 h at RT in a blocking solution of TBS containing 10 % fetal bovine serum, 3 % bovine serum albumin, and 0.01 % Triton X100. They were incubated overnight at 4 °C in rabbit anti-IBA1 antibody (1:1,000 in blocking solution; Wako Pure Chemical Industries) and rinsed in TBS. For immunoperoxidase staining, the sections were then incubated for 1.5 h at RT in goat anti-rabbit IgGs conjugated to biotin (1:200 in blocking solution; Jackson Immunoresearch) and for 1 h with A and B reagents of the ABC Vectastain system (1:100 in TBS; Vector Laboratories). The labeling was revealed using diaminobenzidine (DAB; 0.05 %) and hydrogen peroxide (0.015 %) in TBS for 5 min. For immunogold staining, the sections were incubated with goat anti-rabbit IgGs conjugated to 1.4-nm immunogold (1:50 in blocking solution; Nanoprobes) in TBS overnight at 4 °C. They were rinsed in TBS, then washed twice in 3 % sodium acetate in water. The labeling was revealed using a silver enhancement kit (HQ Silver, Nanoprobes) for 90 seconds at RT. Afterwards, the sections were post-fixed flat in 1 % osmium tetroxide and dehydrated in ascending concentrations of ethanol. They were treated with propylene oxide and then impregnated in Durcupan resin (EMS) overnight at RT. After mounting between ACLAR embedding films (EMS), they were cured at 55 °C for 72 h. Areas of interest (ventral hippocampus CA1 or parietal association areas of the cerebral cortex) were excised from the embedding films and glued on resin blocks.

### N9 microglial cells

N9 microglial cells (provided by Dr. Seguela, Montreal Neurological Institute, Montreal) were cultured in Iscove’s Modified Dulbecco’s Medium (Invitrogen) supplemented with 5 % (*v*/*v*) fetal bovine serum (FBS, Invitrogen) and 1 % (*v*/*v*) penicillin-streptomycin (Invitrogen). Cells were seeded 24 h prior to treatment/media change according to the appropriate density for the indicated assay (lower density for imaging and higher density for biochemical assays). They were maintained at 37 °C with 5 % CO_2_ and >95 % relative humidity.

### DHA/BSA complex preparation

DHA/BSA complex (hereafter referred to as DHA treatment) was prepared by adding 25 mg of DHA (Nu-Check Prep) to ~20 ml of fatty acid-free BSA solution (5 % *w*/*v*, in KRBH buffer; Sigma-Aldrich). The DHA/BSA solution was incubated for 5 h at 37 °C. After the incubation, the pH of the solution was adjusted to 7.4 and the solution was filtered through a 0.22-μm filter. Non-esterified DHA concentration in the solution was determined with the NEFA C method kit (Wako). The final molar ratio of DHA to BSA was approximately 6:1. Aliquots of the stock solution were flushed with argon to prevent oxidation of DHA and were stored at −80 °C. In addition, the presence of BSA was controlled for in those cultures that did not receive the DHA treatment.

### Cells treatment, fixation, and processing for electron microscopy

Cells were treated with DHA (50 μM) with or without LPS (100 ng/ml) for 24 h. The LPS was extracted from *Escherichia coli* of serotype 0111:B4 (Sigma-Aldrich). For control experiments, cells were treated with bovine serum albumin (BSA) at concentrations equivalent to that contained in 50 μM DHA. All chemicals for electron microscopy (paraformaldehyde (16 %; electron microscopy grade), glutaraldehyde (25 %; electron microscopy grade), uranyl acetate, and osmium tetroxide) were purchased from Electron Microscopy Sciences (Fort Washington, PA). Other chemicals were purchased from Sigma (St. Louis MO). N9 cells were seeded in Chamber slides (Lab Tek chamber slides, eight wells per slide Permanox slides, Nunc Inc. Naperville Illinois, USA). Ten thousand cells per square centimeter were grown on surfaces coated with poly D-lysine. After 24-h exposure to the treatments, cell culture medium was removed and replaced with the fixation buffer consisting of 1.5 % paraformaldehyde and 1.5 % glutaraldehyde in 0.2 M cacodylate buffer (pH 7.4). Cells were fixed for 1 h. Following fixation, the cells were carefully washed with 0.1 M cacodylate washing buffer. The washing was repeated three times and cells were post-fixed in 1 % osmium tetroxide. This step was followed by multiple washing in cacodylate buffer; cells were dehydrated and embedded in epon.

### Ultrathin sectioning, TEM imaging, and LB analysis

For studies on brain sections, 70-nm sections containing the ventral hippocampus CA1 radiatum or parietal association areas of the cerebral cortex were generated with a Leica UC7 ultramicrotome. Images were acquired using a FEI Tecnai Spirit G2 microscope equipped with an ORCA-HR digital camera (10 MP; Hamamatsu) operating at an accelerated volatage of 80 kV. For studies involving N9 microglial cells, 100-nm sections were cut using an Ultracut E-ultramicrotome. Images were acquired using a JOEL JEM-2000FX instrument operating at 80 kV. A minimum of 10 cells per experiment were imaged randomly and analyzed qualitatively, with the experimenters blinded to the treatments during imaging and analysis. A semi-quantitative code (+, ++, +++) was used to compare the prevalence of direct contacts between lipid vacuoles or droplets with mitochondria and ER stretches, mitochondrial alterations, and ER dilation, as well the prevalence of filopodia and phagocytic inclusions across experimental conditions. For quantitative analysis, the number of lipid vacuoles and lipid droplets per cell, and their prevalence found within groups (of 2+ vacuoles or droplets) was determined in the four experimental groups. In addition, 50 randomly selected lipid vacuoles per experimental group were traced with the freehand selection tool in ImageJ, and their area was measured.

### Lipidomic analysis

Lipid analysis was performed as previously reported [90]; prior to lipid extraction, internal standards were added corresponding to each lipid class, then lipids were extracted from whole cells by a modified Bligh and Dyer [91] method; samples were dried under nitrogen then resuspended in chloroform. Immediately prior to injection, the extracted lipids were combined with 2:1 methanol/chloroform with 0.1 % (*v*/*v*) ammonium hydroxide. This was injected directly into a Q-TOF 2 mass spectrometer (Waters, Milford, MA) using a nano-esi spray source at 1 μl/min. Spectra were obtained in positive-ion mode (PC + H+, TAG + NH4+) and negative-ion mode (FFA-H+, PA-H+, PE-H+, PG-H+, PI-H+, PS-H+, CL-2H+). Acquired spectra were centroided using the Masslynx software then deconvoluted and deisotoped with excel macros. In our experiments, N9 microglial cells were subjected to lipid extraction followed by ESI-MS to determine acyl chain composition at 0, 3, 9, and 24 h after treatment. Results shown in Fig. 7 are those obtained 24 h after treatment. Control cells were only exposed to BSA in the medium equivalent to the concentration with DHA bound to BSA.

### Phagocytic activity

To measure the extent of phagocytic activity in LPS-activated microglia, N9 microglial cells were treated with fluorescent polystyrene microbeads (FluoSpheres) measuring 1 μm in diameter (Invitrogen #F13083, ex/em = 580/605 nm). Cells were treated with DHA (10–50 μM) with or without LPS (100 ng/ml) for 6 or 24 h. The LPS was extracted from *E. coli* of serotype 0111:B4 (Sigma-Aldrich). For control experiments, cells were treated with BSA at concentrations equivalent to that contained in 25 or 50 μM DHA. Seeding flasks used are 75-cm^2^ cell culture flasks (Sarstedt). FluoSpheres were added during the last 3 h of treatment at a concentration of 1 × 10^6^ particles/mL. Cells were washed and fixed in 4 % paraformaldehyde, labeled with Hoechst 33258 (10 μM, 10 min), mounted on glass slides and imaged using a fluorescence microscope. Four fields were imaged per treatment, and a minimum of 20 cells were imaged per field. The number of cells with internalized FluoSpheres was counted.

### Cytokine measurements

Concentrations of TNFα and IL-1β released from untreated (control) and treated (LPS (100 ng/mL), DHA (50 μM) and DHA + LPS) microglial cells were measured using ELISA (Biolegend). Following treatments (24 h), the samples were appropriately diluted and assessed according to the manufacturer’s protocol. Standard curves were prepared in duplicates for each experiment, and cytokine concentration was calculated from the linear portions of these standard curves as reference. We opted to express increases in TNFα and IL-1β relative to the control values (untreated cells) because these ratios were very consistent and reproducible between repeated experiments. In contrast, the absolute baseline values were dependent on the cell number in three different experiments. On average, the baseline values for TNFα were within low nanomolar range (0.2 to 0.3 ng/mL), whereas the baseline concentrations of IL-1β were much lower picomolar range (20–25 pg/mL).

### Mitochondrial membrane potential

N9 microglial cells were exposed to DHA (5–50 μM) for 24 h. For 6-h treatments, the cells were exposed to DHA (50 μM) in the presence or absence of LPS (0.1 μg/mL). Following treatment, the cells were incubated with tetramethylrhodamine ethyl ester (TMRE, Thermo Fisher, 50 nM) for 20 min, after which the media was refreshed and cells were imaged under a fluorescence microscope. Four fields were imaged per treatment. Intracellular fluorescence was analyzed using ImageJ.

### Cell viability assays

N9 microglial cell death was measured using the Alexa Fluor 488 Annexin V/Dead Cell Apoptosis Kit (Thermo Fisher), according to the recommendations of the manufacturer. Cells were treated with increasing concentrations of DHA (5, 25, 50, 100 μM) in the presence or absence of LPS (0.1 μg/mL) for 24 h, then labeled with Hoechst 33258 (10 μM), Alexa 488-labeled Annexin V and propidium iodide for 15 min. Cells were imaged using a high-throughput fluorescence microscope (Operetta, Perkin Elmer). Five fields were imaged per well, and cells were analyzed using the Columbus software (Perkin Elmer). Cell viability measured by cell counting was performed following treatment with increasing concentrations of DHA (5, 25, 50, 100 μM) in the presence or absence of LPS (0.1 μg/mL) for 24 h. Cells were then labeled with Hoechst 33258 (10 μM, 10 min) and imaged using a high-throughput fluorescence microscope. Five fields were imaged per well, and cell counting was done in Columbus.

### Oxidative stress assay

N9 microglial oxidative stress was measured using the reactive oxygen species indicator CM-H2DCFDA (Thermo Fisher). Cells were loaded with CM-H2DCFDA (10 μM, 30 min), then exposed to increasing concentrations of DHA (10, 25, 50 μM) in the presence or absence of LPS (0.1 μg/mL) for 1 h. Hydrogen peroxide (20 μM) was included as a positive control. Following treatment, intracellular fluorescence was imaged in live cells using a fluorescence microscope. Four fields were imaged per treatment, and 30 cells were analyzed per field.

### Statistical analyses

All experiments were performed at least twice and all samples were analyzed in triplicates. Data are expressed as mean ± SEM and analyzed by ANOVA using Tukey’s and Dunnett’s post hoc test for multiple comparisons. Significant differences are indicated by *p < 0.05, **p < 0.01, and ***p < 0.001.

## Additional files

Additional file 1: Figure S1. Viability of microglial cells exposed to DHA treatment. A–B. Viability of N9 microglial cells in response to DHA (5–100 μM) after 24 h, in the presence or absence of LPS (100 ng/mL). Following treatment, cells were labeled with Hoechst 33258 and incubated with Alexa 488-labeled Annexin V (A) or propidium iodide (B) for 15 min, after which they were imaged under a high-throughput fluorescence microscope. Five fields were imaged per well, and cells were analyzed using the Columbus software. Shown are mean percentages of total cells positive for Annexin V or propidium iodide ± SEM from three independent experiments. **p* < 0.01. C. Viability of N9 microglial cells treated with DHA (5–100 μM) as determined by cell counting after 24 h, in the presence or absence of LPS (100 ng/mL). Following treatment, cells were labeled with Hoechst 33258 and imaged under a high-throughput fluorescence microscope. Five fields were imaged per well, and three wells were included per treatment. The number of nuclei was counted using the Columbus software. Shown are mean percentage values ± SEM as compared to the untreated controls from three independent experiments. **p* < 0.01. (TIF 2392 kb) Additional file 2: Figure S2. Microglial accumulation of lipid vacuoles in aged mouse and human hippocampus. A. Microglia (m) stained for IBA1 with immunoperoxidase showing an accumulation of LBs and lipofuscin granules, various signs of oxidative stress (e.g., dilation of the endoplasmic reticulum; er), as well as direct contacts with dendritic spines (s) and axon terminals (t), in the hippocampus CA1 of an aged mouse (20 months). B. We also initiated studies with human brain to establish if there are similar organelle remodeling processes in the human hippocampus. The key observation is that human microglia, and especially those found nearby blood vessels which are considered to be more active than parenchymal microglia, accumulate lipid droplets of various sizes and electron densities. Example of microglia stained for IBA1 with immunoperoxidase in a middle-aged individual (45-year-old man), observed within the hippocampus. Several lipid droplets of various sizes and differing in electron densities (asterisks) can be seen in the inset (C). Note the small size and grouped organization of several of these droplets. (TIF 11343 kb) Additional file 3: Supplemental methods. (DOCX 93 kb) Additional file 4: Figure S3. Microglial cell morphology, phagocytosis, and mitochondrial alterations following stimulation with LPS. A–B: Microglial cell showing nuclear budding (arrowhead), a phenomenon suggestive of ongoing mitosis, and an accumulation of mitochondria with various alterations (m; inset). In particular, mitochondria partially to completely devoid of cristae and partially to completely devoid of a double external membrane can be seen. The adjacent microglial cell is representative of the most frequently observed morphological phenotype following treatment with LPS, showing several lipid vacuoles (v), similarly to what has been described previously. er = endoplasmic reticulum. C–D: microglial cell showing an accumulation of small mitochondria often devoid of cristae (inset). Budding from larger mitochondria can sometimes be seen (arrowhead). g = golgi apparatus. E–F: Microglial cell containing mitochondria of various sizes, from small to large, and often showing alteration (inset), as well as a large phagocytic inclusion (in). To be noted, only mitochondria showing alteration are annotated in the three insets. (TIF 18662 kb) Additional file 5: Figure S4. Mitochondrial membrane potential of microglial cells exposed to DHA. A. Change in mitochondrial membrane potential in N9 microglial cells exposed to DHA and LPS for 6 h. Mitochondrial membrane potential of N9 microglial cells treated with DHA (50 μM) for 6 h. Following treatment, the cells were incubated with TMRE (50 nM) for 20 min, after which the media was refreshed and cells were imaged under a fluorescence microscope. Three fields were imaged per treatment, and ten cells were imaged per field. Shown are average fold increase in intracellular relative fluorescence intensities ± SEM as compared to untreated control (set to 1) from two independent experiments. **p* < 0.01; ***p* < 0.001 B. Change in mitochondrial membrane potential in N9 microglial cells exposed to DHA (5–50 μM) for 24 h. Following treatment, cells were incubated with TMRE (50 nM) for 20 min, after which the media was refreshed and cells were imaged under a fluorescence microscope. Four fields were imaged per treatment, and two cells were imaged per field. Shown are average fold increase in intracellular relative fluorescence intensities ± SEM as compared to untreated control (set to 1) from three independent experiments. Cells imaged in the absence of TMRE and cells treated with FCCP were negative controls. **p* < 0.01. (TIF 1214 kb) Additional file 6: Figure S5. Reduction of oxidative stress in microglial cells treated with DHA. Oxidative stress in N9 microglial cells following treatment with DHA (10, 25, 50 μM), in the presence or absence of LPS (100 ng/mL) for 1 h. Hydrogen peroxide (20 μM) was included as a positive control. N9 microglial cells were loaded with CM-H2DCFDA (10 μM, 30 min), then exposed to treatment. Following treatment, intracellular fluorescence was imaged in live cells using a fluorescence microscope. Four fields were imaged per treatment, and 30 cells were analyzed per field. Shown are average relative intracellular fluorescence ± SEM (fold increase of control set to 1) from three independent experiments. ***p* < 0.001 compared to control; ##*p* < 0.001 compared to LPS. (TIF 1344 kb) Additional file 7: Figure S6. Phagocytosis of microglial cells exposed to BSA and LPS. Effect of BSA on N9 microglial phagocytic activity with and without LPS. N9 microglial cells were cultured in the presence or absence of BSA (concentration equivalent that of 25 or 50 μM DHA) and LPS (100 ng/mL) for 24 h. FluoSpheres (10^6^ particles/mL) were added to the cells 3 h before the end of treatment. Cells were fixed using 4 % paraformaldehyde and labeled with Hoechst 33258 (10 μM, 10 min). Cells were mounted on glass slides and imaged under a fluorescence microscope. Four fields were imaged per treatment. The number of cells with internalized FluoSpheres was counted. Shown are average percentages of total cells containing FluoSpheres ± SEM from three independent experiments. (TIF 800 kb) Additional file 8: Figure S7. Nuclear fragmentation (between arrowheads) following treatment with LPS. n = nucleus. (TIF 8550 kb)

## Abbreviations

DAB: diaminobenzidine
DHA: docosahexaenoic acid
EPA: eicosapentaenoic acid
ER: endoplasmic reticulum
IBA1: ionized calcium binding adaptor molecule 1
IL-1β: interleukine-1 β
IL-6: interleukine-6
IL-10: interleukine-10
JNK: c-Jun N-terminal kinase
LB: lipid bodies
LPS: lipopolysaccharide
NO: nitric oxide
PS: phosphatidyl serine
TEM: transmission electron microscopy
TNFα: tumor necrosis factor α
