# Supplementary figures and images for: Remodeling of lipid bodies by docosahexaenoic acid in activated microglial cells
Source: J Neuroinflammation. 2016 May 24;13:116. doi: 10.1186/s12974-016-0580-0 (PMC4879742; doi:10.1186/s12974-016-0580-0)

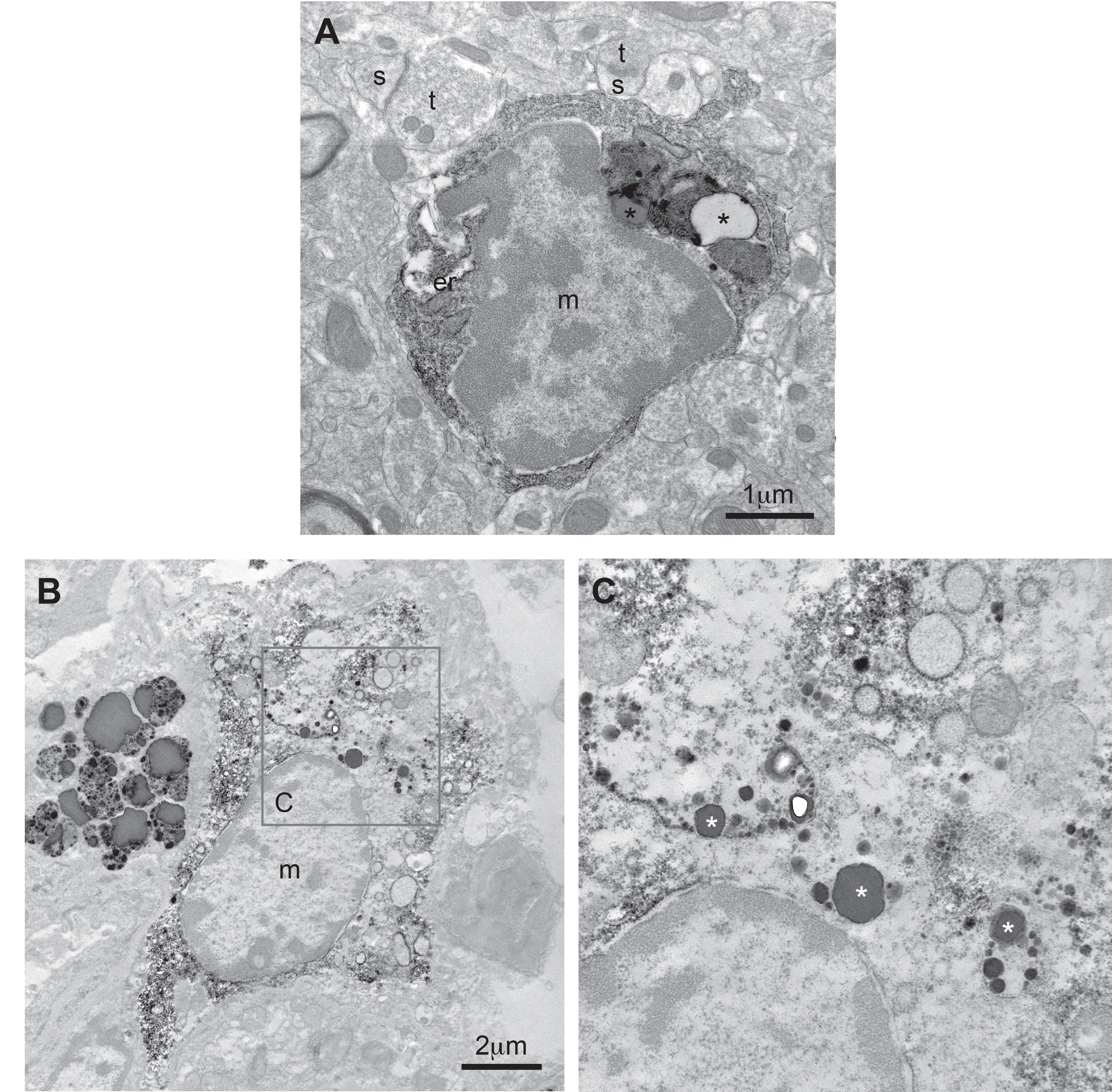

Supplement: Additional file 2: Figure S2. — Microglial accumulation of lipid vacuoles in aged mouse and human hippocampus. A. Microglia (m) stained for IBA1 with immunoperoxidase showing an accumulation of LBs and lipofuscin granules, various signs of oxidative stress (e.g., dilation of the endoplasmic reticulum; er), as well as direct contacts with dendritic spines (s) and axon terminals (t), in the hippocampus CA1 of an aged mouse (20 months). B. We also initiated studies with human brain to establish if there are similar organelle remodeling processes in the human hippocampus. The key observation is that human microglia, and especially those found nearby blood vessels which are considered to be more active than parenchymal microglia, accumulate lipid droplets of various sizes and electron densities. Example of microglia stained for IBA1 with immunoperoxidase in a middle-aged individual (45-year-old man), observed within the hippocampus. Several lipid droplets of various sizes and differing in electron densities (asterisks) can be seen in the inset (C). Note the small size and grouped organization of several of these droplets. (TIF 11343 kb) [file 12974_2016_580_MOESM2_ESM.tif]

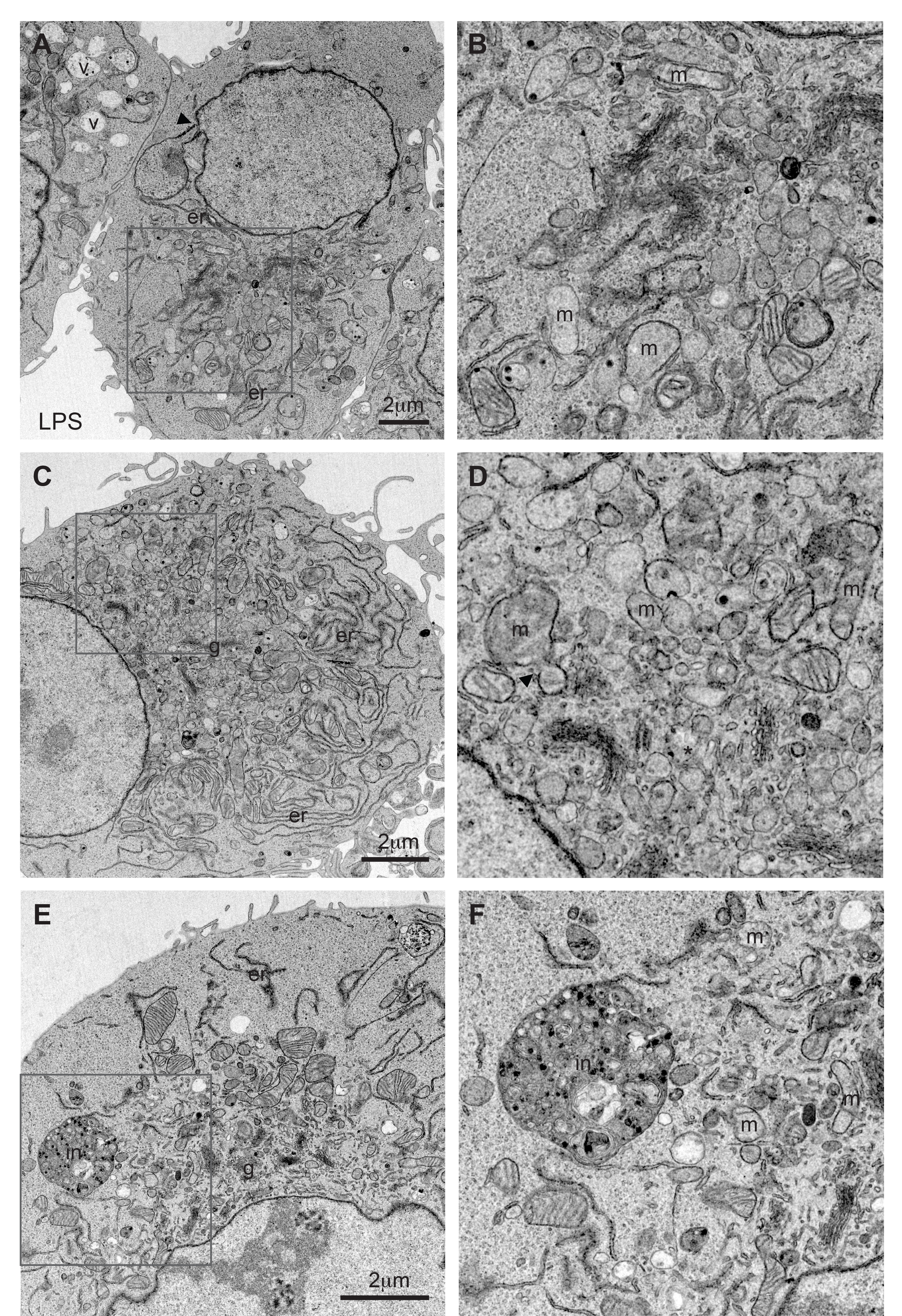

Supplement: Additional file 4: Figure S3. — Microglial cell morphology, phagocytosis, and mitochondrial alterations following stimulation with LPS. A–B: Microglial cell showing nuclear budding (arrowhead), a phenomenon suggestive of ongoing mitosis, and an accumulation of mitochondria with various alterations (m; inset). In particular, mitochondria partially to completely devoid of cristae and partially to completely devoid of a double external membrane can be seen. The adjacent microglial cell is representative of the most frequently observed morphological phenotype following treatment with LPS, showing several lipid vacuoles (v), similarly to what has been described previously. er = endoplasmic reticulum. C–D: microglial cell showing an accumulation of small mitochondria often devoid of cristae (inset). Budding from larger mitochondria can sometimes be seen (arrowhead). g = golgi apparatus. E–F: Microglial cell containing mitochondria of various sizes, from small to large, and often showing alteration (inset), as well as a large phagocytic inclusion (in). To be noted, only mitochondria showing alteration are annotated in the three insets. (TIF 18662 kb) [file 12974_2016_580_MOESM4_ESM.tif]

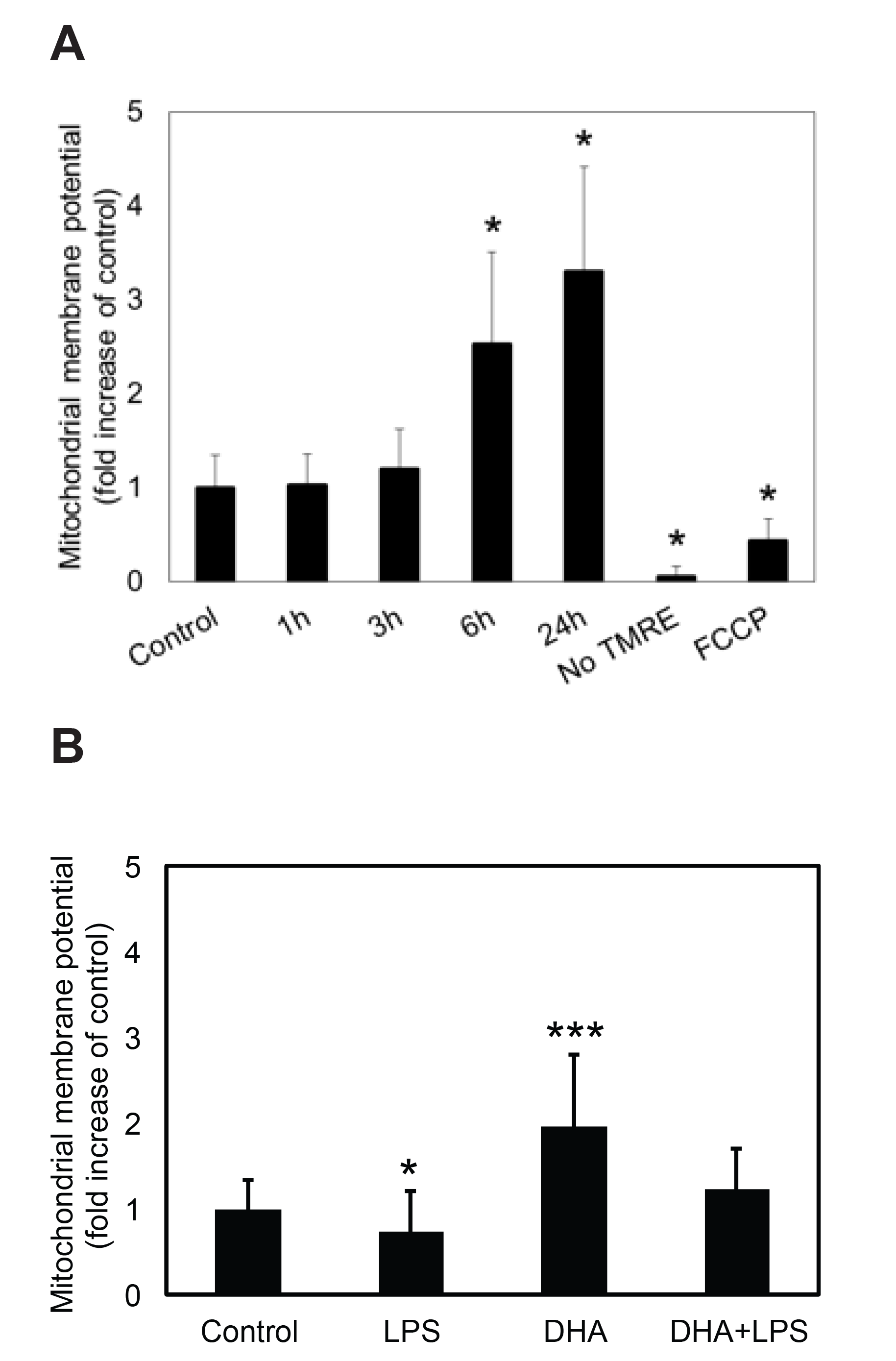

Supplement: Additional file 5: Figure S4. — Mitochondrial membrane potential of microglial cells exposed to DHA. A. Change in mitochondrial membrane potential in N9 microglial cells exposed to DHA and LPS for 6 h. Mitochondrial membrane potential of N9 microglial cells treated with DHA (50 μM) for 6 h. Following treatment, the cells were incubated with TMRE (50 nM) for 20 min, after which the media was refreshed and cells were imaged under a fluorescence microscope. Three fields were imaged per treatment, and ten cells were imaged per field. Shown are average fold increase in intracellular relative fluorescence intensities ± SEM as compared to untreated control (set to 1) from two independent experiments. *p < 0.01; **p < 0.001 B. Change in mitochondrial membrane potential in N9 microglial cells exposed to DHA (5–50 μM) for 24 h. Following treatment, cells were incubated with TMRE (50 nM) for 20 min, after which the media was refreshed and cells were imaged under a fluorescence microscope. Four fields were imaged per treatment, and two cells were imaged per field. Shown are average fold increase in intracellular relative fluorescence intensities ± SEM as compared to untreated control (set to 1) from three independent experiments. Cells imaged in the absence of TMRE and cells treated with FCCP were negative controls. *p < 0.01. (TIF 1214 kb) [file 12974_2016_580_MOESM5_ESM.tif]

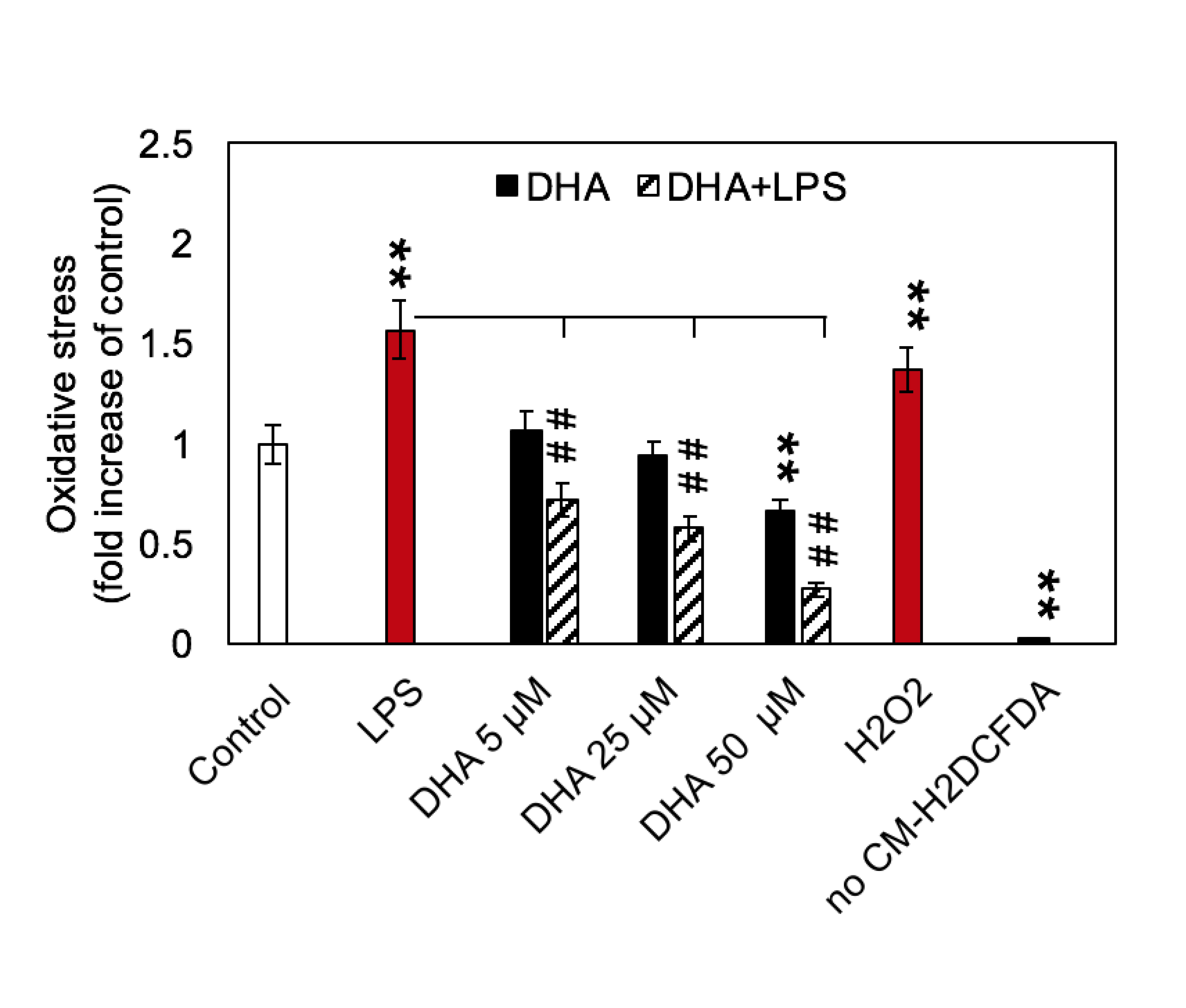

Supplement: Additional file 6: Figure S5. — Reduction of oxidative stress in microglial cells treated with DHA. Oxidative stress in N9 microglial cells following treatment with DHA (10, 25, 50 μM), in the presence or absence of LPS (100 ng/mL) for 1 h. Hydrogen peroxide (20 μM) was included as a positive control. N9 microglial cells were loaded with CM-H2DCFDA (10 μM, 30 min), then exposed to treatment. Following treatment, intracellular fluorescence was imaged in live cells using a fluorescence microscope. Four fields were imaged per treatment, and 30 cells were analyzed per field. Shown are average relative intracellular fluorescence ± SEM (fold increase of control set to 1) from three independent experiments. **p < 0.001 compared to control; ##p < 0.001 compared to LPS. (TIF 1344 kb) [file 12974_2016_580_MOESM6_ESM.tif]

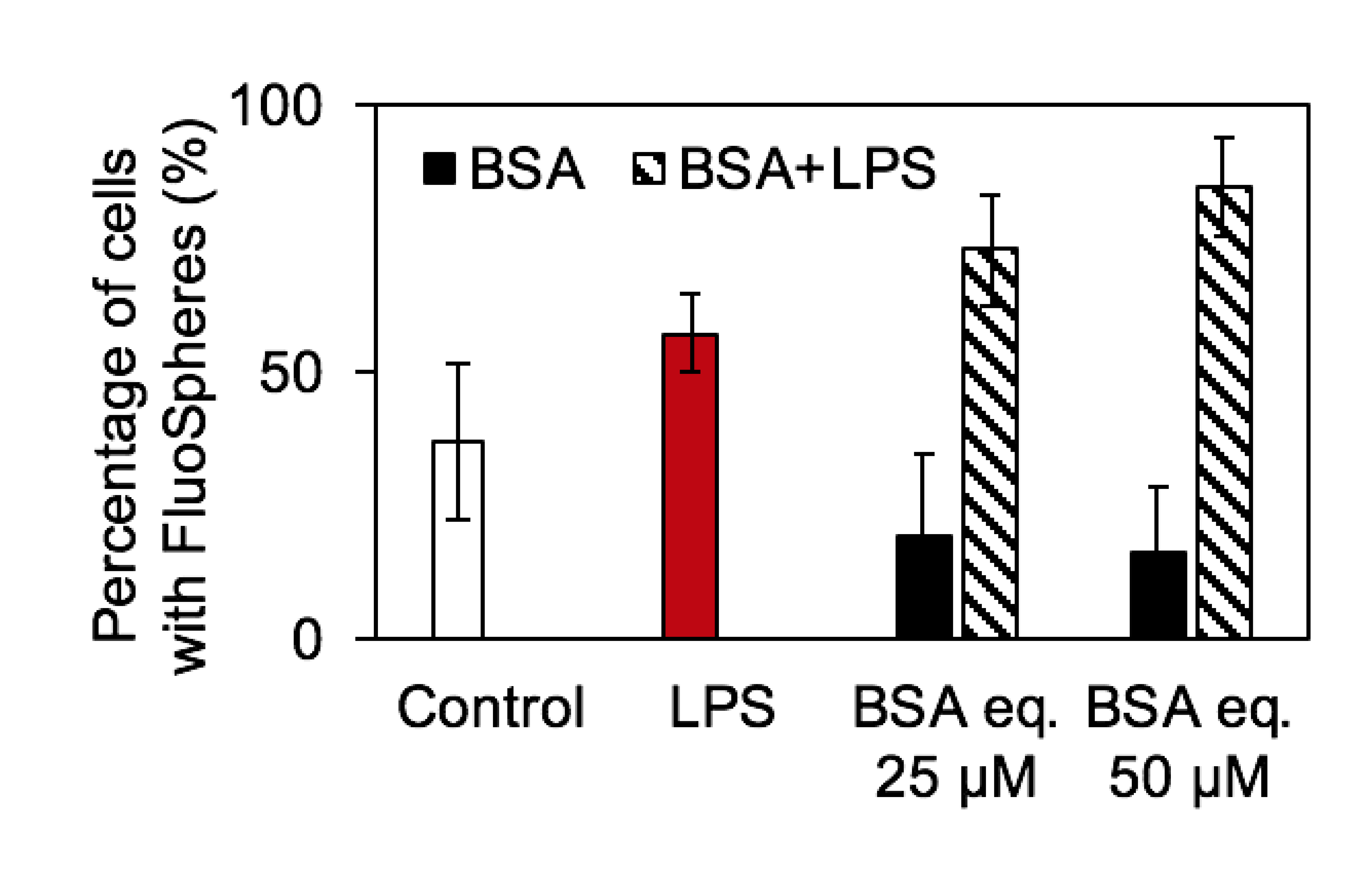

Supplement: Additional file 7: Figure S6. — Phagocytosis of microglial cells exposed to BSA and LPS. Effect of BSA on N9 microglial phagocytic activity with and without LPS. N9 microglial cells were cultured in the presence or absence of BSA (concentration equivalent that of 25 or 50 μM DHA) and LPS (100 ng/mL) for 24 h. FluoSpheres (106 particles/mL) were added to the cells 3 h before the end of treatment. Cells were fixed using 4 % paraformaldehyde and labeled with Hoechst 33258 (10 μM, 10 min). Cells were mounted on glass slides and imaged under a fluorescence microscope. Four fields were imaged per treatment. The number of cells with internalized FluoSpheres was counted. Shown are average percentages of total cells containing FluoSpheres ± SEM from three independent experiments. (TIF 800 kb) [file 12974_2016_580_MOESM7_ESM.tif]

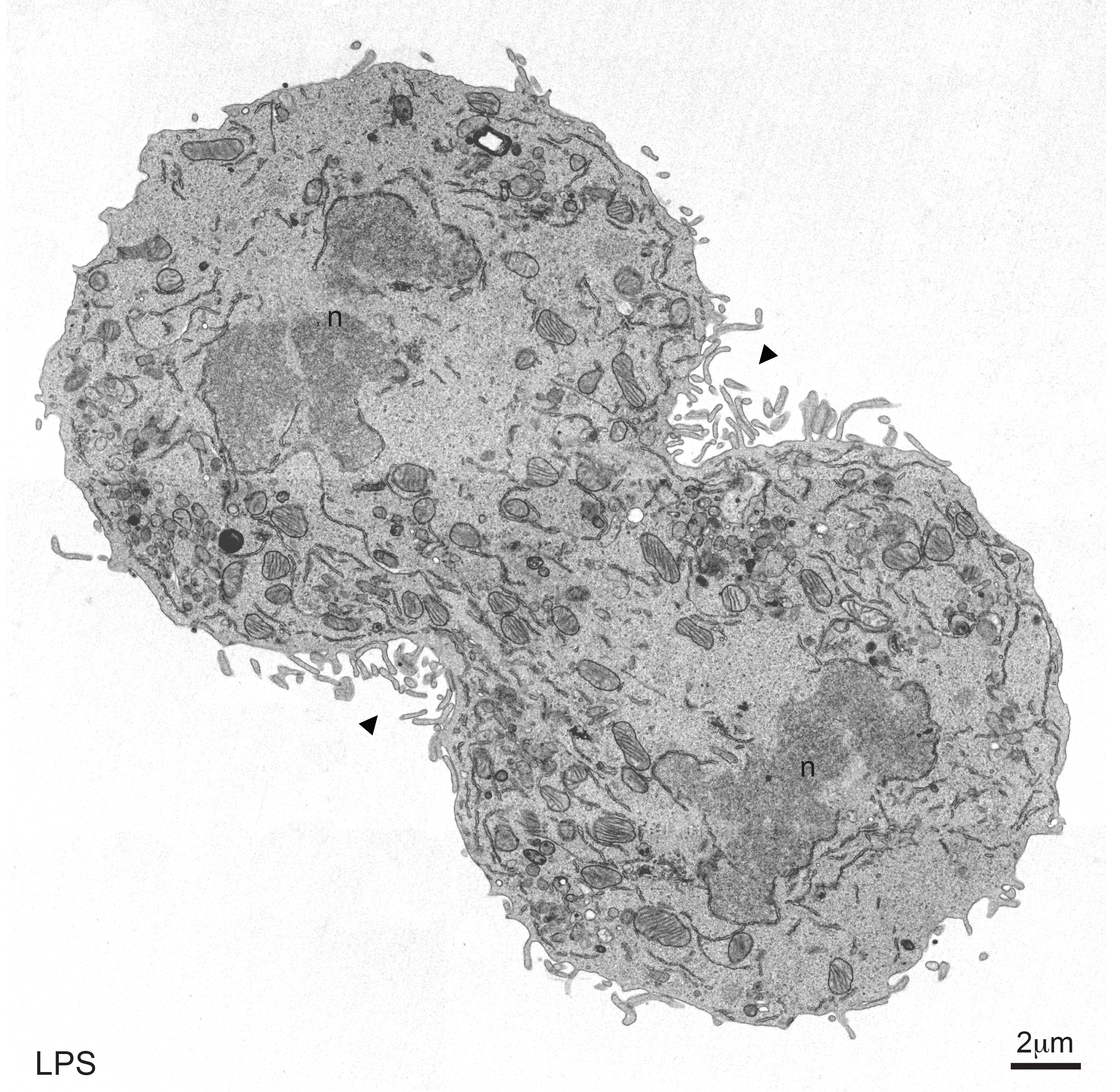

Supplement: Additional file 8: Figure S7. — Nuclear fragmentation (between arrowheads) following treatment with LPS. n = nucleus. (TIF 8550 kb) [file 12974_2016_580_MOESM8_ESM.tif]
