# Supplementary material for: Remodeling of lipid bodies by docosahexaenoic acid in activated microglial cells
Source: J Neuroinflammation. 2016 May 24;13:116. doi: 10.1186/s12974-016-0580-0 (PMC4879742; doi:10.1186/s12974-016-0580-0)
Supplement: Additional file 3: — Supplemental methods. (DOCX 93 kb) [file 12974_2016_580_MOESM3_ESM.docx]

**Supplemental materials**

**Supplemental Methods**

**Human brain samples.** One postmortem brain with a post-mortem delay of 18 hours was obtained from a 45-year old man with no clinical or pathological evidence of neurological or psychiatric disorders. The material was taken from the brain bank established at the *Centre de Recherche de l’Institut Universitaire en santé mentale de Québec* (CRIUSMQ). Brain banking and postmortem tissue handling procedures were approved by the Ethic Committee of the *Institut Universitaire en santé mentale de Québec* and by *Université Laval*. The brain was obtained with written consent and the analyses were performed in conformity with the Code of Ethics of the World Medical Association (Declaration of Helsinki). The brain was first cut in half along the midline and hemi-brains were sliced into 2 cm-thick slabs along the coronal plane. These slabs were fixed by immersion in 4% paraformaldehyde at 4°C for 3 days. They were then stored at 4°C in a 0.1M phosphate-buffered saline (PBS, pH 7.4) solution containing 15% sucrose and 0.1% sodium azide. The slabs containing the hippocampus were then cut with a freezing microtome into 50 µm-thick sections that were serially collected in PBS and stored at –20°C in a solution containing glycerol and ethanediol until immunostaining.

**Mouse brain samples.** 20 months old (n=4) male mice on a C57Bl/6 background were examined as described in the main methods for the purpose of validating microglial accumulation of LBs in aging.
